# A Case Report of Polyneuropathy, Organomegaly, Endocrinopathy, Monoclonal Gammopathy, and Skin Changes (POEMS) Syndrome: A Diagnostic Iceberg

**DOI:** 10.7759/cureus.56229

**Published:** 2024-03-15

**Authors:** Arturo Anticona León, Mauricio G Crovetto Urteaga, Katherin M Plasencia Correa, Wilmer Jara Garcia

**Affiliations:** 1 Neurology Service, Hospital de Alta Complejidad Virgen de la Puerta, Trujillo, PER

**Keywords:** chronic inflammatory demyelinating polyneuropathy (cidp), plasma cell disorder, axonal polyneuropathy, caslteman disease, poems

## Abstract

POEMS syndrome (Polyneuropathy, Organomegaly, Endocrinopathy, Monoclonal plasma cell disorder, Skin changes) refers to a rare paraneoplastic entity linked to a plasma cell disorder, characterized by multiple systemic manifestations that must be studied together to establish a timely diagnosis. We report a case of a 60-year-old female who had been initially classified to have Guillain Barré syndrome for one year and seven months, receiving three cycles of immunoglobulin without a positive response. The clinical picture was characterized by painful paresthesias in four limbs and paraparesis, with the patient also developing distal cutaneous hyperpigmentation and multiple adenopathies. Neuroconduction studies revealed chronic sensorimotor axonal polyneuropathy and albumin-cytological dissociation was evidenced in the study of cerebrospinal fluid (CSF). In the subsequent evaluation, Lambda light chains and a lymph node biopsy compatible with Castleman’s disease were found, and hence it was determined that the patient met the criteria for POEMS syndrome. This case report highlights the importance of incorporating other diagnostic perspectives when encountering patients with polyneuropathy of immunological origin who fail to respond to conventional therapies.

## Introduction

The POEMS syndrome (Polyneuropathy, Organomegaly, Endocrinopathy, Monoclonal plasma cell disorder, Skin changes) is defined as a rare phenotype of a multisystem autoimmune paraneoplastic condition [1]. It has a global incidence of 0.15 per 100,000 inhabitants with a 1-1.9 per 100,000 prevalence rate. It is associated with a mortality rate of 3% and generates sequelae in 60% of cases; 20% of cases are disabling in nature [2]. The syndrome has a female predilection in the Japanese population, with a female-to-male ratio of 2.5/1. It predominantly affects individuals between their fifth and sixth decades of life, with an average time to diagnosis of 13-18 months [3]. We discuss a case of a patient with immunologically based polyneuropathy who failed to respond adequately to conventional treatment and was eventually diagnosed with POEMS syndrome.

## Case presentation

The patient was a 60-year-old female with arterial hypertension and hypothyroidism as underlying pathologies; her clinical symptoms had begun one year and seven months ago, characterized by painful dysesthesia in the lower limbs, which had been managed as venous insufficiency. She had gone to a general hospital as her condition had not gotten better and eventually worsened, causing ascending weakness and paresthesias in her lower limbs that had initially restricted her ability to walk. Eventually, the upper limbs had also been affected.

During her first hospitalization, the neuroconduction studies had shown an absence of nerve conduction potentials in the median, ulnar, motor peroneal, superficial peroneal, and sural nerves. Needle electromyography showed positive waves, repetitive discharges, and fasciculations in the lower limbs with the absence of voluntary activation compatible with axonal sensory-motor polyneuropathy. The patient had been initially diagnosed with Guillain Barré syndrome. After physicians used immunoglobulin to treat her, there had been an apparent improvement. She returned to the same hospital after two months due to a relapse and worsening, and persistent protein-cytological dissociation had been discovered during a cerebrospinal fluid (CSF) analysis. It had been approached as a chronic inflammatory demyelinating polyneuropathy (CIDP) for which a new cycle of immunoglobulin had been administered. Two months later, she was readmitted to the hospital due to persistence and lack of improvement and given a third cycle of immunoglobulin without clinical changes.

**Figure 1:**
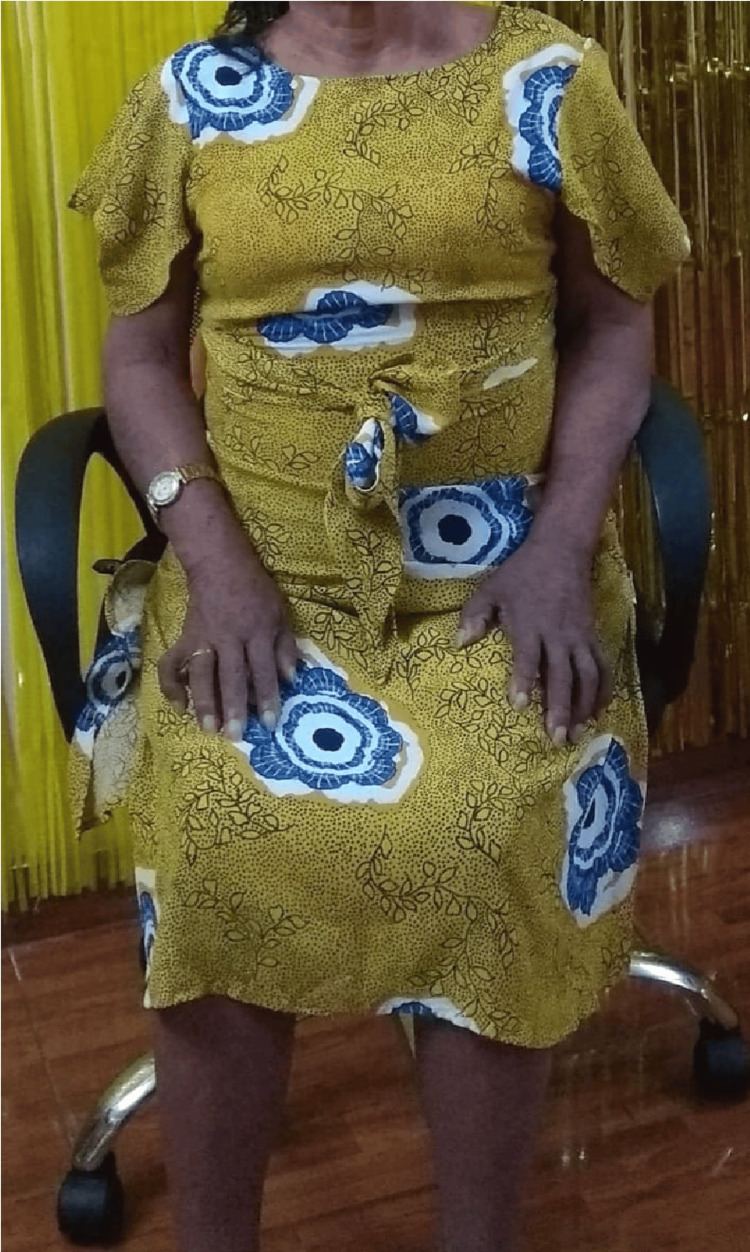
Image showing hyperchromic spots distributed mid-distal in the extremities

The patient was then referred to our institution and hospitalized in the neurology service. The examination revealed edema and intermediate-distal hyperpigmentation in all extremities (Figure 1), multiple lymphadenopathy, areflexic quadriparesis, and bilateral papilledema. Her condition was studied as a paraneoplastic syndrome. From the tests performed, the following laboratory results were highlighted (Table 1): parameters included polyglobulia, protein/cytological dissociation, increased interleukin 6 and prolactin levels, and an increase in the Gamma fraction linked to Lambda light chains in the electrophoretic proteinogram with immunofixation. Electromyography and nerve conduction velocity were compatible with chronic axonal motor sensory polyneuropathy.

**Table 1:** Laboratory results

Type of test	Result	Reference values
Cerebrospinal fluid: proteins	116 mg/dl	<45 mg/dl
Cerebrospinal fluid: glucose	60 mg/dl	50-90 mg/dl
Cerebrospinal fluid: cell count	0	<10 cells/mm^3^
Interleukin 6 (IL-6)	10.89 pg/ml	0.49-3.45 pg/ml
Thyroxine (T4)	0.734 mg/dl	0.93-1.71 mg/dl
Thyroid-stimulating hormone (TSH)	5.779 UI/ml	0.27-4.2 UI/ml
Prolactin	39.14 ng/ml	2.5-23.1 ng/ml
Albumin	3.08 g/dl	3.4-4.8 g/dl
Alkaline phosphatase	452 U/L	65-320 U/L
Hemoglobin	16.8 g/dl	12-15 g/dl
Electrophoretic proteinogram	Gamma: 1742 mg/dl	70-1600 mg/dl
Immunofixation	IgG heavy chains associated with Lambda light chain	

**Figure 2:**
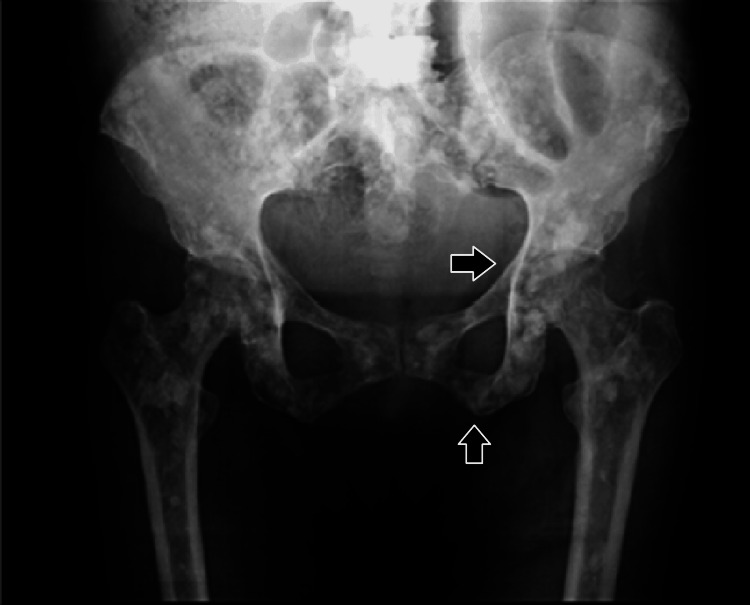
Skeletal survey showing osteolytic lesions in pelvic bones

**Figure 3:**
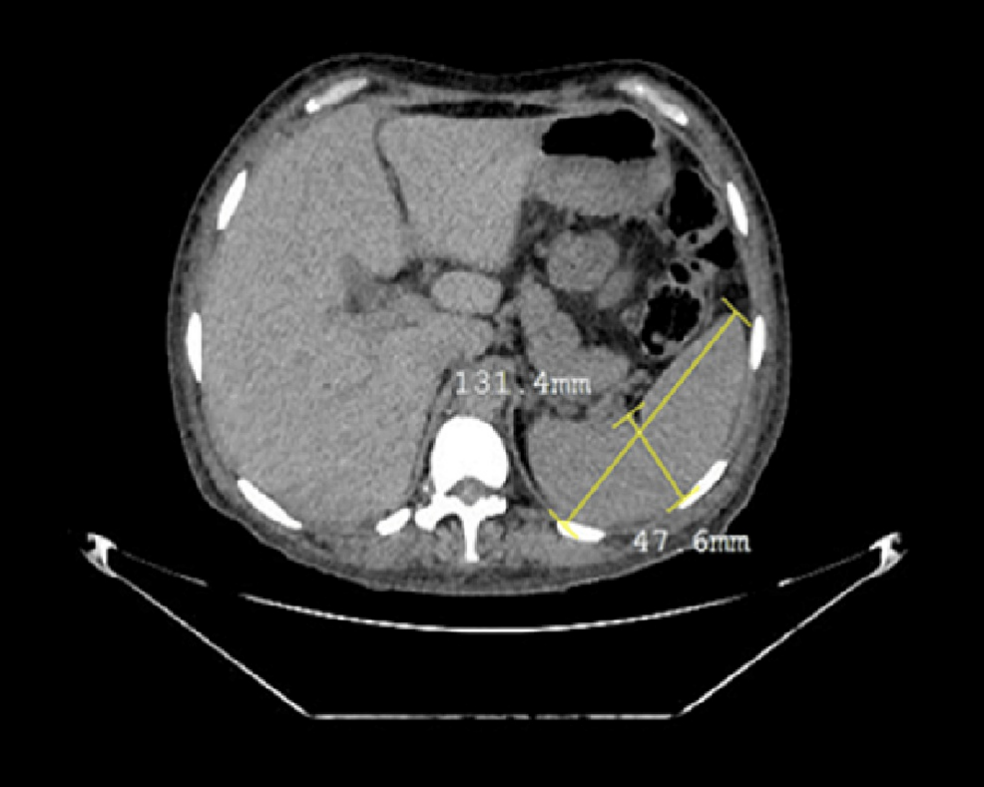
Abdominopelvic CT indicating splenomegaly The dimensions of the patient's spleen were 14.17 x 13.14 x 4.76 cm, and the splenic index measured 886.3 cm^3 ^(a value >440 cm^3^ suggests splenomegaly [4] CT: computed tomography

The cervical ultrasound showed reactive lymph nodes in the cervical region and atypical cervical lymph nodes. In the thyroid, there was a solid nodule in the left lobe and heterogeneous nodules with calcifications in the left lobe (TIRADS 4). During the skeletal study, blast lesions were observed in the parietal region of the cranium, pelvis, and femur (Figure 2), and CT studies showed hepatosplenomegaly and multiple blast lesions in bone structures with multiple lymphoproliferative lymphadenopathy (Figure 3).

Due to the high clinical suspicion and to rule out clonal disorder of plasma cells, a lymph node biopsy with immunohistochemistry was performed, the pathological report revealed the following findings: lymph nodes with numerous lymphoid follicles in the cortex and medulla, the majority presenting involuted germinal centers with vascular hyalinization with lymphocytes from the mantle zone arranged in concentric rings (onion layer); and interfollicular zones with increased high endothelial venules, with the presence of plasma cells. Immunohistochemistry showed CD3 positivity in T lymphocytes, CD20 positivity in B lymphocytes (highlighting follicles of different sizes), CD21 positivity in follicular dendritic cells, CD138 positivity in interfollicular plasma cells, CD23 positivity in follicular dendritic cells, and BCL2 positivity in lymphocytes of the area of the mantle, and these findings were determined to be compatible with the hyaline-vascular variant of Castleman's disease. The bone marrow biopsy showed involvement by plasma cell neoplasia associated with grade 2 focal marrow fibrosis. The patient was transferred to the hematology service to start chemotherapy, which led to improvement in her physical appearance, achieving skin depigmentation, and gaining weight. She also recovered the mobility of her lower limbs and was able to walk again. The follow-up with the patient has been maintained.

## Discussion

POEMS syndrome is a chronic, progressive multisystem disorder with a complex pathogenesis that requires a multidisciplinary approach; it is associated with an average survival of 13 years, which varies depending on its clinical manifestations [3]. Due to its intrinsic complexity, diagnostic confusion with other pathologies of immunological origin such as CIDP is common, which is one of the main barriers to the correct management of this entity.

Due to the variability in clinical manifestations, the Mayo Clinic has established diagnostic criteria for POEMS syndrome; and patients have to fulfill two mandatory criteria, at least one major criterion, and one minor criterion to be diagnosed with this condition (Table 2).

**Table 2:** Diagnostic criteria for POEMS syndrome The diagnosis of POEMS syndrome is confirmed when both of the mandatory major criteria, one of the three other major criteria, and one of the six minor criteria are present [5] ^a^Adrenal, thyroid, pituitary, gonadal, parathyroid, and pancreatic ^b^Hyperpigmentation, hypertrichosis, glomeruloid hemangiomata, plethora, acrocyanosis, flushing, white nails ^c^Splenomegaly, hepatomegaly, or lymphadenopathy ^d^Edema, pleural effusion, or ascites POEMS: Polyneuropathy, Organomegaly, Endocrinopathy, Monoclonal plasma cell disorder, Skin changes; VEGF: vascular endothelial growth factor

Mandatory criteria	Major criteria	Minor criteria
Clonal plasma cell disorder, neuropathy	Sclerotic bone lesions, increased serum VEGF levels, Castleman's disease.	Endocrine disorders^a^, skin disorders^b^, organomegaly^c^, thrombocytosis/polycythemia, extravascular volume overload^d^, papilla edema

The peak in incidence occurs between 50 and 60 years of age [3,6], as in our patient. Clinically, neuropathy is the predominant characteristic; it is usually bilateral ascending, symmetrical, with sensory-motor involvement, with pain being the main symptom in 10-15% of cases [5], which aligns with our case. The patient was initially managed as CIDP, ignoring the clinical characteristics such as lymphadenopathy, hyperpigmentation, hepatosplenomegaly, areflexia, gait alterations, and peripheral edema that are suggestive of POEMS syndrome [7], some of which could be observed in the patient during the clinical examination following her admission to our service; the organomegaly was confirmed by CT and the adenopathy by ultrasound.

It is recommended that any patient with a diagnosis of chronic inflammatory polyneuropathy who fails to respond adequately to the usual treatment be investigated for POEMS syndrome and the following studies should be carried out, which have the best cost/effectiveness ratio: levels of vascular endothelial growth factor (VEGF), evaluation of bone alterations and bone marrow biopsy [5]. In our case, the patient was repeatedly subjected to immunoglobulin cycles to which she responded poorly, in addition to the delay in the suspicion of another etiological entity with subsequent progression of the disease. Unfortunately, VEGF levels could not be quantified since the institution is not equipped to conduct this study. The CSF study and neuroconduction studies constitute key tests in the evaluation of all patients with polyneuropathy of unknown origin and those in whom CIDP is suspected. The findings usually reveal protein-cytological dissociation and typically the conduction velocity is reduced with marked early secondary axonal loss, respectively [8]. In this case, a protein/cell ratio of 116/0 was found in CSF. In the neuroconduction study, a polyneuropathy with a pattern of chronic axonal damage was found, a mandatory criterion for this syndrome.

Monoclonal Immunoglobulinemia is another criterion; IgA or IgG are usually detected and, in most cases, lambda chains as well [9]. In our patient, an increase in IgG associated with the Lambda light chain was found, and plasma cell neoplasia was evident in the bone marrow biopsy. Between 11 and 30% of POEMS patients who have a documented clonal plasma cell disorder also have documented Castleman's disease or Castleman-like histology [5]. In one series, 25 of the 43 biopsied nodes were diagnosed with Castleman's disease and 84% of these were of the hyalinevascular type [10]. This is a major criterion, which was confirmed with the lymph node biopsy performed on our patient. Endocrinopathy is a central but poorly understood feature of POEMS. In one series, approximately 84% of patients had a recognized endocrinopathy, with hypogonadism as the most common endocrine abnormality, followed by thyroid abnormalities, glucose metabolism abnormalities, and finally adrenal insufficiency [11]. Our patient had low T4 and high TSH, compatible with clinical hypothyroidism.

The papilledema found in the patient constitutes another of the minor criteria for the diagnosis of POEMS syndrome. In a series of 94 patients, it was found in 50%, in addition to polycythemia, which can be found in 12-19% of cases [5]. The reported case met the required criteria for the diagnosis of POEMS syndrome since it fulfilled two mandatory criteria, a major criterion, and five minor ones.

## Conclusions

Clinically, polyneuropathy can be attributed to multiple etiologies, ranging from a primary pathology of the nervous system to that secondary to other diseases. This case report highlights the importance of the role of the clinical unit in the diagnostic assessment. By viewing the various signs and symptoms together as a single entity, the most accurate diagnosis can be reached, which can lead to the prompt initiation of treatment to improve the patient's quality of life.
